# Anatomical Entity Recognition with a Hierarchical Framework Augmented by External Resources

**DOI:** 10.1371/journal.pone.0108396

**Published:** 2014-10-24

**Authors:** Yan Xu, Ji Hua, Zhaoheng Ni, Qinlang Chen, Yubo Fan, Sophia Ananiadou, Eric I-Chao Chang, Junichi Tsujii

**Affiliations:** 1 State Key Laboratory of Software Development Environment, Key Laboratory of Biomechanics and Mechanobiology of Ministry of Education, Beihang University, Beijing, China; 2 Microsoft Research Asia, Beijing, China; 3 The National Centre for Text Mining, School of Computer Science, The University of Manchester, Manchester, United Kingdom; University of Memphis, United States of America

## Abstract

References to anatomical entities in medical records consist not only of explicit references to anatomical locations, but also other diverse types of expressions, such as specific diseases, clinical tests, clinical treatments, which constitute implicit references to anatomical entities. In order to identify these implicit anatomical entities, we propose a hierarchical framework, in which two layers of named entity recognizers (NERs) work in a cooperative manner. Each of the NERs is implemented using the Conditional Random Fields (CRF) model, which use a range of external resources to generate features. We constructed a dictionary of anatomical entity expressions by exploiting four existing resources, i.e., UMLS, MeSH, RadLex and BodyPart3D, and supplemented information from two external knowledge bases, i.e., Wikipedia and WordNet, to improve inference of anatomical entities from implicit expressions. Experiments conducted on 300 discharge summaries showed a micro-averaged performance of 0.8509 Precision, 0.7796 Recall and 0.8137 F1 for explicit anatomical entity recognition, and 0.8695 Precision, 0.6893 Recall and 0.7690 F1 for implicit anatomical entity recognition. The use of the hierarchical framework, which combines the recognition of named entities of various types (diseases, clinical tests, treatments) with information embedded in external knowledge bases, resulted in a 5.08% increment in F1. The resources constructed for this research will be made publicly available.

## Introduction

Since anatomical locations play a crucial role in organizing information and knowledge in the clinical domain, the identification of expressions which refer to them in text has been identified as an important target of Natural Language Processing (NLP) in the clinical domain. In this paper, we use the term *anatomical entities* to refer to expressions which correspond to anatomical locations such as body parts, organs, and their subparts. Such expressions may be explicit or implicit. Earlier studies on anatomical entity recognition focused only on expressions which refer to explicit anatomical entities [1]–[5]. However, implicit references are abundant in clinical records. Indeed, clinical experts can hardly realize whether expressions are explicit or implicit [6]. For example, a clinical record may report that a patient has had an ECG test. Whilst the term “*ECG*” itself does not refer to an anatomical entity explicitly, the mention of an ECG test does suggest to an expert that the patient has problems with his or her heart.

In this research, entities such as ECG are defined as implicit anatomical entities. Although such entities belong to different semantic classes from anatomical entities, they are nonetheless strongly associated with anatomical entities (e.g. heart). Mentions of such implicit anatomical entities are as important as those of explicit anatomical entities, since they provide clinical experts with clues about patients' conditions with respect to specific anatomical locations. Although several tools have been developed for clinical information extraction, none of them has focused on the recognition of implicit anatomical entities.

Recognition of implicit anatomical entities presents two challenges with respect to current technology. Firstly, since implicit entities themselves belong to diverse semantic classes, expressions which refer to them appear in different contexts, depending on their semantics classes. Thus, we cannot construct one single recognizer which assumes that they appear in homogeneous local contexts. Secondly, to determine which semantic classes correspond to implicit anatomical entities requires the domain knowledge of clinical experts.

In order to identify implicit anatomical entities, we have developed a hierarchical framework, in which different layers of named entity recognizers (NERs) work in a cooperative manner. In order to resolve the first problem, we used as the first layer of NER an existing tool capable of recognizing multiple semantic classes, i.e., the multi-class recognizer which we developed for I2B2 challenge tasks [7]. This tool recognizes three major classes of entities (i.e., Diseases, Clinical Tests and Clinical Treatments) that could potentially constitute implicit references to anatomical entities. The second NER layer recognizes explicit anatomical entity mentions, whilst the third layer determines which of the candidate entities from the first layer actually represent implicit references to anatomical entities. All layers of the framework are based on the Conditional Random Field (CRF) models [8]–[10].The third layer exploits Wikipedia and WordNet as knowledge resources. Entities recognized by the multi-class recognizer (first level) are checked against the knowledge resources. If the resources specify an explicit link exists between the candidate implicit entity and a specific anatomical entity, then specific features used by the CRF model are set.

A comprehensive dictionary of expressions is known to improve the performance of named entity recognizers. We have thus supplemented the use of the above external resources with the construction of a dictionary of known anatomical entity expressions using a number of existing resources, i.e. the Unified Medical Language System (UMLS) [11], Medical Subject Headings (MeSH) [12], RadLex [13] and table of BodyParts3D [14]. The dictionary matching results are used as features by both the explicit and implicit anatomical entity recognizers.

Abbreviations, which are abundant in clinical records, are one of the major causes of difficulties for NERs used within clinical applications. This is because a large proportion of abbreviations occurring in clinical records are local and ad hoc in nature. i.e., they are only used in a given text and their full forms appear in the same text. Due to their local nature, we cannot include them in a dictionary. Instead, we assume that their full forms can be discovered using existing abbreviation detection techniques [7], [15]. Since abbreviation detection is not the focus of this research, we make use of coreference chains that are already annotated in our corpus to find the full forms of abbreviations. Each abbreviation is replaced by its full form prior to the application of the NERs. The coreference feature is explained in greater detail in the *Methods* section.

## Related Work

Named entity recognition is the first step of information extraction (IE), which maps information in text to the knowledge of a domain. The Medical Language Extraction and Encoding System (MedLEE) was one of the earliest systems developed to carry out named entity extraction on clinical text. Their extraction method used is based on the use of semantic lexicons and hand-written rules [16]–[19]. Hripcsak [16] found that NLP has a potential to extract clinically important information from narrative reports, as an aid to automated decision-support and clinical research. Friedman [18] further improved extraction of relevant clinical information and UMLS coding using NLP. In 2009 and 2010, the I2B2 challenge tasks [20], [21] constituted the first serious attempt of focus attention on named entity extraction in the clinical domain. The tasks included extraction of medical concepts (problem, treatment and test). Since the I2B2 organizers provided a reasonably large annotated corpus, most of the groups participating in the I2B2 challenge tasks used machine learning-based approaches. In particular, state-of-the-art NER performance has been achieved by systems based on the Conditional Random Field (CRF) model. Recent trends in NER and ER (Event Recognition) in the biomedical domain is surveyed by Ananiadou et al. [22].

There have been a number of efforts to building dictionaries of anatomical entities and associated ontologies. In 2003 and 2008, Rosse et al. [23], [24] proposed a fundamental model of anatomy and proposed a reference ontology of anatomy. While MedLEE performs anatomical entity extraction [25], [26], the underlying dictionary does not link to any reference ontology. In 2010, Naderi et al. [27] presented the organism tagger, focusing on recognizing various subcategories of organisms. Their system is a hybrid rule-based/machine learning system. Machine learning-based anatomical entity recognition has been studied by Pyysalo et al. [6], and they constructed a dictionary using resources available in the open biomedical ontologies (OBO) repository. However, these previous studies dealt only with explicit anatomical entities listed in the reference ontology; they have not exploited information embedded in external resources to identify implicit anatomical entities.

In the general domain of NERs, there have been numerous attempts to use external resources to improve the performance of systems. For example, Kazama et al. [28] explored the use of Wikipedia as such an external knowledge base. Cucerzan [29] also used Wikipedia to disambiguate named entities in a large-scale system for texts in the general domain. In the clinical domain, Rink et al. [30] used Wikipedia to produce features for relation classification among medical concepts, and achieved the best performance in the relation extraction task of the 2010 I2B2 challenge. Xu et al. [31] used diverse external resources (e.g., Wikipedia, WordNet, Probase) to produce features for co-reference recognition, and achieved the best performance in the coreference task of the 2011 I2B2 challenges. Xu et al. [32] also used web resources to improve their sentiment classifier in the 2011 I2B2 sentiment analysis challenge. The present work is a natural extension of the previous attempts at anatomical entity recognition. In particular, our work focuses on how to use relational information embedded in ontological resources, such as entities and their anatomical locations.

## Methods

In order to examine how anatomical entities are referred to in clinical records (i.e., discharge summaries in this study), we first asked a clinical expert to annotate expressions which he considered to be “anatomical entities”. As a result, we found that a large number of expressions annotated as anatomical entities did not explicitly refer to anatomical locations. Therefore, we decided to distinguish such implicit entities from explicit anatomical entities in our annotation scheme. These two types of anatomical entities are also treated separately by our entity recognizer.

**Figure 1:**
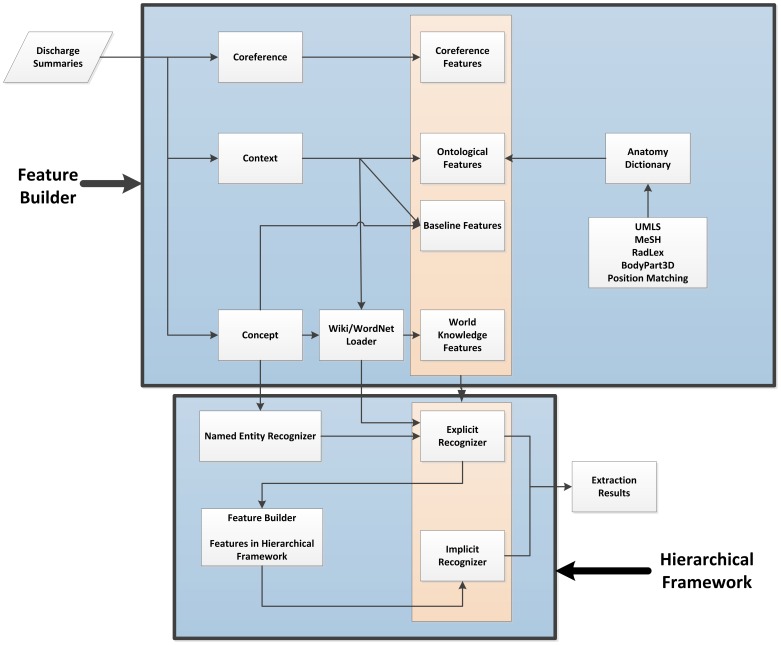
Overview of system workflow.

The architecture of the system is shown in **Figure 1**. As is common practice in NERs, we apply standard language NLP tools (i.e., POS tagging, parsing, and character string processing) to extract features which have been found effective in NERs operating in other domains. We refer to these commonly-used standard set of features as the baseline features.

In addition to the baseline features, the second layer CRF recognizer uses features derived from the first layer recognizer, in addition to external knowledge sources (i.e., Wikipedia [33] and WordNet [34]), which we will discuss in detail in the following sections.

### Annotation

Consistency and comprehensiveness of annotation greatly affect the performance of the system and the credibility of experimental results. In order to ensure the quality of annotation, we performed several iterations of preliminary annotation prior to the final annotation effort.

Our annotated dataset is the same set of 300 discharge summaries used by the I2B2 challenges, which consists of 28642 sentences. The final annotated corpus includes 16690 explicit anatomical entity tokens and 5564 implicit anatomical entities tokens. The link is following: https://drive.google.com/file/d/0B1A1rRX4lVdxbmhUVFUyWlRQOFk/edit?usp=sharing.

Annotation was performed by three annotators, two with a biomedical engineering background and one with a clinical background.

### Annotation guidelines

Expressions of anatomical location in biomedical text are often categorized into one of five different levels, i.e., systems, organs, tissue, cells, chemicals (e.g. ions and molecules) [35]. Since cells and chemicals commonly exist in every part of the human body and are not useful for the current study, we only annotated expressions referring to the top three levels: systems, organs, and tissue.

An explicit anatomical entity is defined as an expression which directly denotes a specific body component of the system, organ, or tissue level. In other words, we consider the medical domains which can describe the human body at such levels as explicit anatomical entities in clinical texts. Such explicit anatomical entities are not limited to nouns or noun phrases. Adjectives or adjectival phrases such as “pulmonary” are also treated as explicit entities.

Implicit anatomical entities comprise a wide range of medical terms. In this study, medical terms that belong to the following categories are defined as potential implicit anatomical entities: (1) Medical problems (e.g., diseases) which occur in specific parts of the body or are caused by abnormalities of specific body components. For example, “pneumonia” implicitly refers to the lung, while “Hypertension” implicitly refers to vessels, as it is mostly physiologically caused by blood vessel abnormalities, such as narrowing of arteries and arterioles. (2) Clinical treatments specifically aimed at certain body components, such as “mastectomy”. (3) Clinical tests that are closely related to body components such as “ECG”.

Apart from expressions belonging to these three classes, expressions were also annotated as implicit anatomical entities if they express relations with body components or contain useful clinical information. For example, the set of adjectives which have structures of “positional prefix or word+body component” were also annotated. Expressions of this type refer to one or more peripheral areas around the body component, such as “supraclavicular” and “infraclavicular”.

According to their corresponding full forms, abbreviations are treated as either explicit or implicit. If the full form of an abbreviation contains an explicit anatomical entity, it is annotated as explicit. For example, “cp” (chest pain), is identified as an explicit anatomical entity. If an explicit anatomical entity is not included in the full form, then the abbreviation will be treated as an implicit entity.

**Figure 2:**
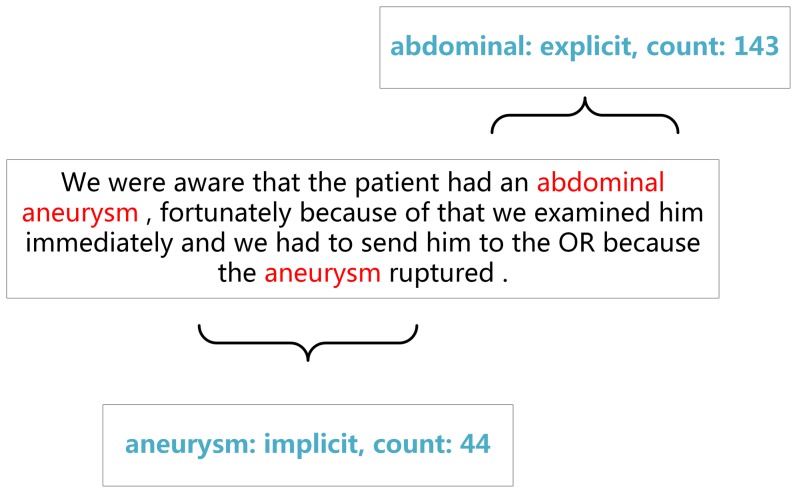
Annotation samples.

Note that words like “neurology” are associated with components of human body but do not refer to specific components. Thus, we do not annotate them as anatomical entities. Special attention is given to different usages of the same terms. For example, “visual” mostly refers to the observer (e.g., visual inspection) but it can be used to denote an anatomical entity of the patient. While the former usage is not annotated as an anatomical entity, the latter is. **Figure 2** shows the example of annotations in discharge summaries.

### Annotation Flow

The three annotators annotated 10 discharge summaries independently of each other and then discussed differences among their annotations. Based on the results of discussion, each annotator independently produced their own set of guidelines. The next round of annotation was performed independently based on these individual sets of guidelines. The same cycle of discussion, revisions of individual guidelines, and independent annotation was repeated until reasonable convergence of annotations was reached. The three annotators then compiled their individual sets of guidelines into a unified set of guidelines.

Using thee unified guidelines, a further two rounds of annotation were performed. The first round was carried out by the two annotators (A1 and A2) with the biomedical engineering background, independently of each other. The annotations produced by A1 and A2 were checked by the third annotator (A3) with the clinical background. If there was a disagreement between A1 and A2, A3 took the role of adjudicator and explained his judgment to A1 and A2. The guidelines were further revised based on the outcome of this process. The final version of the guidelines was used in the annotation of the complete set of 300 discharge summaries. Both A1 and A2 performed the annotation work independently on the whole set of discharge summaries, while A3 made the final decision in case of disagreements between A1 and A2.

### Inter-annotator agreement

We used the kappa coefficient [36] to measure the inter-annotator agreement. **Table 1** summarizes the inter-annotator agreement between A1 and A2. **Table 2** shows the inter-annotator agreement between each annotator and the gold standard. The gold standard constitutes the corpus following adjudication by A3 on the differences between the annotations of A1 and A2.

**Table 1:** Inter-annotator agreement between A1 and A2.

	Explicit	Implicit
True positive	13011	3287
False positive	951	491
False negative	522	312
k	0.9284	0.8812

**Table 2:** Inter-annotator agreement between each annotator and the gold standard.

		Explicit	Implicit
A1	True positive	13787	3577
	False positive	1495	465
	False negative	924	302
	k	0.8901	0.8937
A2	True positive	13116	3328
	False positive	1679	596
	False negative	972	389
	k	0.8768	0.8590

As shown in **Table 1** and **Table 2**, some level of disagreement still existed between A1 and A2 during the final annotation stage. However, the differences were very small. The gold standard may still contain annotation errors, since adjudication by A3 was performed only when A1 and A2 gave different annotations. However, considering the small k between A1 and A2, the remaining errors in the gold standard are expected to be very few in number, and the adjudicated corpus is accurate enough to be used in practice.

In the following experiments, we use the gold standard as the training and the test data sets using five-fold cross-validation.

### CRF Model and Features

As mentioned above, we employed the CRF model in this work due to its wide and successful application in other NER tasks. As the baseline features, we used a standard set of features which have been found useful in previously reported NERs of diverse types. Subsequently, we added a set of features specific to explicit and implicit anatomical entities. As illustrated in Figure 1, the system consists of two layers of NERs, which are both trained using the CRF model.

An NER based on the CRF model sees the entity recognition problem as a sequence labeling problem. Each recognizer assigns one of the three labels Begin/Inside/Outside (BIO) to each word in a sentence. B and I labels, also called B-tag and I-tag, mean that the corresponding word constitutes beginning or intermediate word of a named entity, respectively. An O-tag means that the word does not constitute part of a named entity. A CRF model assigns one of these tags to each word in a sentence, successively from left to right, by observing the word itself and the local context in which the word appears. A word and its local context are represented by features attached to both the focused word and the words in its context. The performance of a CRF-based recognizer is determined by the set of features which are used to characterize words. **Table 3** shows the features used in our system, which are explained in detail in the following sections.

**Table 3:** List of features in this task.

Category	Features
Baseline features	Original
	Capital Upper
	Upper
	Normalized form
	Prefix and Suffix
	Concept dictionary matching
	Concept type
	Stanford Parser POS
	Enju Parser POS
Ontological features	4 dictionaries matching (DF1)
	Position matching(DF2)
Coreference features	Coreference dictionary matching (CF)
World knowledge features	Wiki word matching (WF1)
	Wiki word matching (WF2)
	Wiki word matching (WF3)
Hierarchical feature	Hierarchical feature(HF)

#### Baseline features

The baseline features are those which have been commonly used in entity recognizers in previous studies. These features are computed by using standard NLP tools. They are:

1. Original word feature: the word itself.
2. Capital upper feature (Binary feature): 1 if the initial character of the word is an upper case letter, otherwise 0.
3. Upper case feature (Binary feature): 1 if all characters in the word are upper case letters, otherwise 0.
4. Part of Speech (POS) feature: the POS of a word (noun, verb, adjective, preposition, etc.), as determined by the Enju parser [37] and Stanford parser [38].
5. Original forms of name entities are used as a feature obtained by the basement of Enju parser.
6. Prefix and Suffix features: used to allow recognition of morphological variants of words to be mapped to a normalized form. For example, “abdominal” (abdomen). The prefix feature was derived from combinations (in order) of the first eight characters in a word and suffix feature was derived from combinations (in order) of the last eight characters in a word.

**Figure 3:**
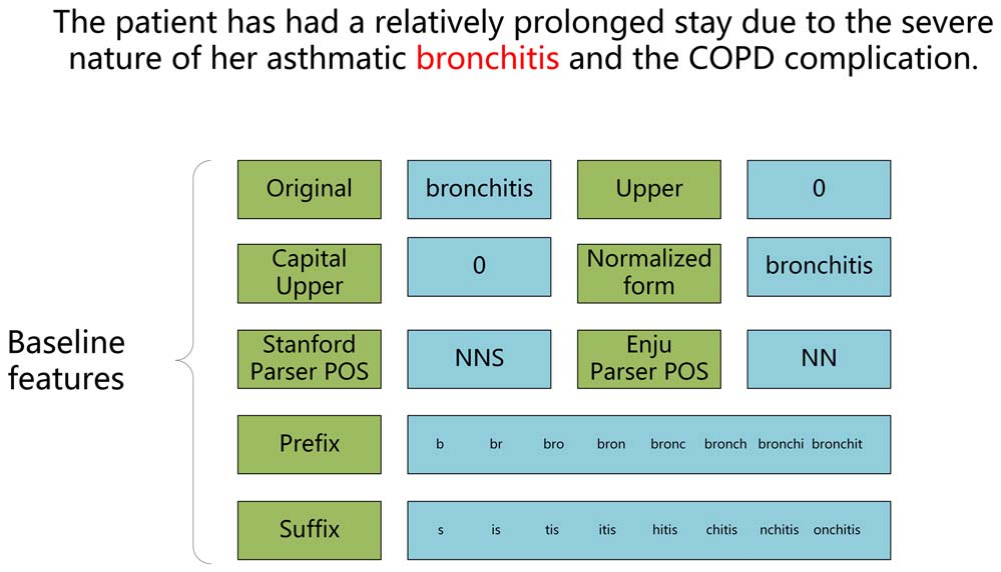
Detailed explanation of baseline features.

**Figure 3** gave a detailed explanation of baseline features for the named entity “bronchitis”, which can express the standard feature formation in CRF model.

#### Ontological features: DF1 and DF2

Due to the ambiguity of named entity expressions, existence of nested expressions, and incompleteness of dictionaries, the performance of a recognizer solely based on dictionary matching is known to be unsatisfactory. However, it is also known that the results of dictionary matching against a comprehensive resource as features of a CRF-based recognizer can be very effective for improving the performance of the recognizer. That is, an ontological feature of a word is set to 1 when the word appears as part of a word sequence which matches an entry in a dictionary.

We prepared two dictionaries to compute ontological features (DF1 and DF2) used in our recognizer. The effectiveness of such an ontological feature largely depends on the comprehensiveness of the dictionary. To construct a comprehensive dictionary of anatomical entities, we have used four resources: UMLS, MeSH, RadLex, and BodyPart3D. The first dictionary (Dictionary-1) was constructed by extracting relevant entries from each of these four resources (further details are provided below). Dictionary-1 is expected to cover explicit anatomical entities. As **Table 4** shows, the actual coverage of Dictionary-1 is much higher than any one of the four individual resources. The coverage in this table refers to the percentage of expressions annotated as explicit named entities in the gold standard dataset which appear in each resource. DF1 is the ontological feature based on this dictionary.

**Table 4:** Numbers of entities in dictionaries.

Dictionary	Number of explicit tokens matched in dictionary	Coverage of explicit named entities
**UMLS**	3012	18.05%
**MeSH**	2174	13.03%
**RadLex**	3238	19.40%
**BodyParts3D**	1595	9.56%
**Total without duplications (Dictinary-1)**	4019	24.08%

In Dictionary-1, 77504 entities are extracted from UMLS, belonging to the semantic types “bpoc” (body part and organ), “tisu” (tissue), “blor” (body location or region) and “bsoj” (body space or junction). From MeSH (Medical Subject Headings), 622 entities belonging to relevant categories are extracted. We extracted all entities (a total of 11406) classified under the type “anatomy_metaclass” in RadLex. From BodyPart3D, 1524 anatomical entities were extracted. We have removed duplications of entities extracted from these resources. As a result, Dictionary-1 contains 86,002 entities in total.

Dictionary-2 contains positional adjectives or adjectival phrases, which can be combined with anatomical entity expressions to create larger units of anatomical expressions. DF2 is the feature associated with this dictionary.

Positional words and phrases may be combined with anatomical expressions to produce new anatomical expressions. The position matching dictionary (Dictionary-2) contains of total of 43 such expressions and 1524 entities. Since the set of such expressions is a closed one, we enumerated them by manual inspection of discharge summaries. Dictionary-2 contains words such as “left” (left arm), “bilateral” (bilateral knees) and “distal” (distal ulnar). Abbreviations of positional words were also included, such as “bilat” (bilateral). The dictionary contributes to the accuracy of boundary detection of anatomical expressions (e.g. “bilateral knees” and “left hand” are recognized as anatomical expressions, instead of “knee” and “hand”).

#### Coreference features

Coreference chains were exploited to alleviate the problem caused by abbreviations. If an abbreviation and its full name appear in the same discharge summary, the abbreviation is called a *local abbreviation*. That is, a local abbreviation is introduced in the current text. As explained previously, the nature of local abbreviations means that we cannot provide a dictionary of them in advance. However, there have been several research efforts focusing on coreference of local abbreviations and their full forms [7], [15]. These works have showed that coreference relations between local abbreviations and their full forms can be recognized with relatively high accuracy. In the current work, instead of implementing these algorithms, we used coreference links already provided in the gold standard I2B2 corpus. If a coreference chain contains at least one expression recognized as an anatomical entity, the co-reference feature of all expressions in the chain is set to 1. Local abbreviations are treated in the same way as their full forms if the full forms are anatomical entities.

#### World knowledge features: WF1, WF2, and WF3

For implicit anatomical entity recognition, we have to solve two separate problems. The first problem is to identify a set of entities that belong to other semantic classes (diseases, clinical tests, clinical treatments, etc.) but are strongly associated with specific anatomical entities. Since there are no dictionaries that define which members of the above semantic classes correspond to implicit anatomical entities, we cannot use simple dictionary matching as we do for explicit anatomical entities (i.e. DF1).

The second problem is identification of the boundaries of implicit entities. Because implicit entities themselves belong to different semantic classes, the contexts in which they appear differ, depending on their semantic classes. A CRF recognizer which treats implicit entities as a single class would not be able to recognize them accurately.

The second problem leads to the two-layer architecture of our system. Instead of applying a single CRF model directly, we first apply the multi-class CRF recognizer [35], which we developed for I2B2 challenge tasks. The multi-class recognizer identifies spans entities belonging to three different classes (i.e., diseases, clinical tests, and clinical treatments). We refer here to the named entities extracted by this recognizer as medical concepts. These named entities constitute implicit anatomical entity candidates.

To solve the first of the above problems, we use two knowledge sources, i.e., Wikipedia and WordNet, to try to determine where a link exists between the medical concept and an explicit named entity. Since neither Wikipedia nor WordNet is a structured knowledge base, they do not express structured associations between implicit entities and explicit entities. Instead, they just provide free text definitions of medical concepts. Thus, we have to judge whether these free text concept definitions imply associations with specific anatomical entities. In the current system, we use simple heuristics for this judgment. That is, we check whether the definition of a medical concept includes any anatomical entity appearing in the Anatomy Dictionary (Dictionary-1). If so, the medical concept is taken represent an implicit anatomical entity. In WordNet, the complete definition of the concept is considered, while in Wikipedia, we treat the first three sentences of the entry for the concept as the definition.

These two steps, i.e., the application of the multi-class entity recognizer and recognition of candidates for implicit anatomical entities from the external resources, can be seen as a sophisticated dictionary matching process for implicit anatomical entities. That is, entities recognized by the multi-class recognizer are checked with corresponding entries in Wikipedia and WordNet to see whether they are associated with entries in the anatomy dictionary (Dictionary-1). If such associations are found, the features (WF2 for Wikipedia and WF3 for WordNet) of component words of the entries are set to 1. In the same way as the ontological feature of DF1 for explicit anatomical entities, these features are used as features of the CRF recognizers for both explicit entities and implicit entities. Since many expressions used in discharge summaries are not formal medical terms (e.g. ex for extremity), they do not have corresponding entries in Wikipedia. In order to alleviate such mismatches between expressions in discharge summaries and entries in Wikipedia, we used another feature, WF1, as explained below.

**Wiki word matching (WF1):** Regardless of the results of the first layer of the CRF recognizer, we consider all the words in a discharge summary sentence (except for stop words, such as “the”, “and”, “in”, etc.) and, if the word has a corresponding entry in Wikipedia, we check whether any words in the definition appear in Dictionary-1. If so, the WF1 feature of the word is set to 1, otherwise the feature is set to 0.

**Wiki word of concept matching (WF2):** For this feature, instead of considering all the words in discharge summaries, we consider only those corresponding to medical concepts (as determined by our multi-class CRF recognizer). For each medical concept word, we determine whether there is a corresponding entry in Wikipedia. If so, we check whether the definition matches any entries in Dictionary-1. If so, the feature WF2 of all the words in the expressions are set to 1, otherwise 0. Note that named entity expressions recognized by the first layer recognizer may consist of more than one word.

**WordNet word of concept matching (WF3):** We take named entity expressions recognized as such by the first layer CRF recognizer, and, if there is a corresponding entry in WordNet, we check whether the any words in the words in definition appear in Dictionary-1. If so, the feature WF2 of all the words in the expressions will be set to 1, otherwise 0.

#### A hierarchical framework for implicit anatomical entity recognition

Our framework approaches the problem of recognizing anatomical entities using two separate CRF recognizers, one for explicit anatomical entities and the other for implicit entities. The system consists of the following three steps:

**[Step 1]** Recognition of entities of belonging three semantic classes (Diseases, Clinical Tests and Treatments), which may constitute implicit anatomical entities. This step is carried out by our multi-class CRF recognizer (first layer recognizer) developed for I2B2 challenges.
**[Step 2]** Recognition of explicit anatomical entities by the explicit anatomical entity recognizer (second layer recognizer).
**[Step 3]** Recognition of implicit anatomical entities by the implicit anatomical entity recognizer (third layer recognizer).

As input to the recognizers in **[Step 2]** and **[Step 3]**, the system computes several types of features, i.e. baseline features, dictionary-based features (DF1 and DF2), knowledge-based features (WF1, WF2 and WF3) and a hierarchical feature (HF). To compute HF, we apply the explicit anatomical entity recognizer (**[Step 2]**) to definitions in Wikipedia. Specifically, the first three sentences in every entry from Wikipedia were extracted and used as test data for the explicit anatomy entity recognizer. We can judge the result of hierarchical feature by seeing if any explicit named entity was recognized. Unlike other features, the hierarchical feature (HF) is only used to recognize implicit anatomical named entities.

**Figure 4:**
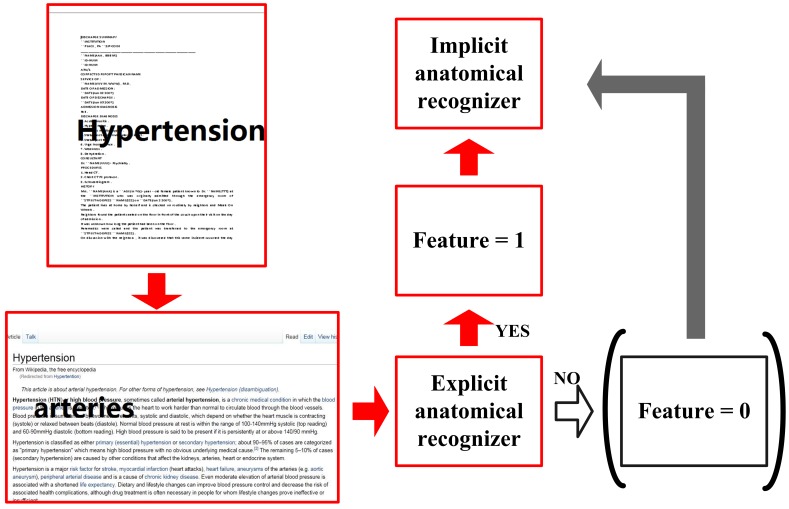
Building the hierarchical feature for implicit anatomical recognizer.

**Figure 4** illustrates how the hierarchical feature (HF) is built into the framework. “Arteries”, an explicit entity, appears in the definition of “Hypertension” in Wikipedia, and is recognized by the explicit anatomical entity recognizer (**[Step 2]**). Thus the feature value of HF for the word “hypertension” is set to “1”. This feature contributes to the negative judgment by the explicit anatomical entity recognizer and the positive judgment by the implicit anatomical entity recognizer.

The recognizers in **[Step 2]** and **[Step 3]** are trained using 5-fold cross-validation on the annotated corpus. Since the corpus also contains annotations of diseases, clinical tests and treatments (as annotated by the I2B2 organizers), we compute the features of WF2 and WF3 for entities annotated as such by the I2B2 organizers. These computed features are used for training the recognizers. For testing purposes, we performed two experiments, one using the I2B2 gold standard annotation and the other using the results of the multi-class recognizer (**[Step 1]**), to compute WF2 and WF3.

## Experiments

In order to evaluate the effect of features and the size of training data, we conducted six groups of experiments, split into two sets. In order to evaluate our novel methods independently of the performance of the multi-class recognizers developed for I2B2, we firstly conducted preliminary experiments using the gold standard I2B2 annotated text as our source of diseases, clinical tests and treatment annotations. We then carried out a second set of experiments, in which the multi-class recognizer was used to recognize diseases, clinical tests and treatments (**[Step 1]**). The results of the two different sets of sets of experiments allow us to assess the influence of errors introduced by the multi-class recognizer on anatomical entity recognition.

The performance of system is evaluated using the three standard performance metrics.i.e., precision (P), recall (R) and F1 (F) [39]. We use the strictest criterion for evaluation on boundary detection, which means that both of the right and the left boundaries of anatomical entities must be correctly recognized.

The first group of experiments (using the gold standard disease, clinical tests and treatment annotations from I2B2) was designed to evaluate the effectiveness of each of our resource-derived features. The results are shown in **Table 5**. A further group of experiments in **Table 6** shows the cumulative effects of combining together features on the performance of the recognizers. **Table 7** shows the effect of the training data size on the performance. We varied the size of the test data from 50 to 300 discharge summaries while the training data size was always 240, which was extracted from the 300 summaries. In each experiment, five-fold cross-validation was used to evaluate the performance.

**Table 5:** Performance of individual feature added to baseline using gold standard medical concepts.

	Explicit recognizer				Implicit recognizer				Total	
	P	R	F	INC	P	R	F	INC	Micro F	INC
Baseline	0.9168	0.7839	0.8452	/	0.9206	0.6919	0.7900	/	0.8320	/
Baseline+DF1	0.9088	0.8296	0.8674	0.0222	0.9224	0.7094	0.8020	0.0120	0.8520	0.0200
Baseline+DF2	0.9239	0.7922	0.8530	0.0078	0.9136	0.7005	0.7930	0.0030	0.8385	0.0065
Baseline+CF	0.9200	0.7883	0.8491	0.0039	0.9198	0.7067	0.7993	0.0093	0.8371	0.0051
Baseline+WF1	0.9297	0.8052	0.8630	0.0178	0.9256	0.7205	0.8103	0.0203	0.8503	0.0183
Baseline+WF2	0.9330	0.8001	0.8615	0.0163	0.9483	0.7403	0.8315	0.0415	0.8542	0.0222
Baseline+WF3	0.9301	0.7987	0.8594	0.0142	0.9377	0.7281	0.8197	0.0297	0.8498	0.0178
Baseline+HF	/	/	/	/	0.9466	0.7419	0.8318	0.0418	/	/

*Inc is increment compared with baseline.

**Table 6:** Performance of different feature combinations using gold standard medical concepts.

	Explicit recognizer			Implicit recognizer			Micro	INC
No	P	R	F	P	R	F	F	
Baseline	0.9168	0.7839	0.8452	0.9206	0.6919	0.7900	0.8320	/
DF1	0.9088	0.8296	0.8674	0.9224	0.7094	0.8020	0.8520	0.0200
DF1+DF2	0.9108	0.8439	0.8761	0.9299	0.7160	0.8091	0.8604	0.0284
DF1+DF2+CF	0.9234	0.8469	0.8835	0.9361	0.7248	0.8170	0.8678	0.0358
DF1+DF2+CF+WF1	0.9396	0.8667	0.9017	0.9463	0.7391	0.8300	0.8848	0.0528
DF1+DF2+CF+WF1+WF2	0.9451	0.8735	0.9079	0.9484	0.7415	0.8327	0.8901	0.0581
DF1+DF2+CF+WF1+WF2+WF3	0.9481	0.8776	0.9114	0.9505	0.7455	0.8356	0.8936	0.0616
DF1+DF2+CF+WF1+WF2+WF3+HF	0.9481	0.8776	0.9114	0.9686	0.7631	0.8537	0.8978	0.0658

*Inc is increment compared with baseline.

**Table 7:** Performance with training datasets of various sizes using gold standard medical concepts.

Data Size	Explicit recognizer			Implicit recognizer			Micro	INC
	P	R	F	P	R	F	F	
50	0.9316	0.8245	0.8748	0.9527	0.6364	0.7631	0.8491	/
100	0.9384	0.8483	0.8911	0.9593	0.6966	0.8071	0.8717	0.0226
150	0.9423	0.8621	0.9004	0.9654	0.7147	0.8213	0.8821	0.0330
200	0.9479	0.8713	0.9080	0.9664	0.7418	0.8393	0.8922	0.0431
250	0.9488	0.8743	0.9102	0.9678	0.7595	0.8511	0.8964	0.0473
300	0.9481	0.8776	0.9114	0.9686	0.7631	0.8537	0.8978	0.0487

*Inc is increment compared with baseline.

Finally, we replaced the gold standard I2B2 annotations with the results produced by the multi-class recognizer that we developed for I2B2 challenges. **Table 8**, **Table 9**, and **Table 10** show the results.

**Table 8:** Performance of individual feature added to baseline using automatically predicted medical concepts.

	Explicit recognizer				Implicit recognizer				Total	
	P	R	F	INC	P	R	F	INC	Micro F	INC
Baseline	0.8123	0.7048	0.7545	/	0.8217	0.6282	0.7120	/	0.7443	/
Baseline+DF1	0.8113	0.7461	0.7773	0.0228	0.8236	0.6303	0.7141	0.0021	0.7625	0.0182
Baseline+DF2	0.8268	0.7112	0.7647	0.0102	0.8171	0.6304	0.7117	−0.0003	0.7519	0.0076
Baseline+CF	0.8252	0.7106	0.7636	0.0091	0.8167	0.6298	0.7112	−0.0008	0.7510	0.0067
Baseline+WF1	0.8318	0.7221	0.7731	0.0186	0.8293	0.6502	0.7289	0.0169	0.7624	0.0181
Baseline+WF2	0.8333	0.7215	0.7734	0.0189	0.8459	0.6692	0.7472	0.0352	0.7670	0.0227
Baseline+WF3	0.8314	0.7186	0.7709	0.0164	0.8376	0.6590	0.7376	0.0256	0.7628	0.0185
Baseline+HF	/	/	/	/	0.8447	0.6713	0.7481	0.0361	/	/

*Inc is increment compared with baseline.

**Table 9:** Performance of different feature combinations using automatically predicted medical concepts.

	Explicit recognizer			Implicit recognizer			Micro	INC
No	P	R	F	P	R	F	F	
Baseline	0.8123	0.7043	0.7545	0.8217	0.6282	0.7120	0.7443	/
DF1	0.8105	0.7452	0.7765	0.8233	0.6427	0.7219	0.7636	0.0193
DF1+DF2	0.8116	0.7582	0.7840	0.8304	0.6491	0.7286	0.7710	0.0267
DF1+DF2+CF	0.8256	0.7599	0.7914	0.8362	0.6578	0.7363	0.7784	0.0341
DF1+DF2+CF+WF1	0.8495	0.7781	0.8122	0.8452	0.6687	0.7467	0.7967	0.0524
DF1+DF2+CF+WF1+WF2	0.8487	0.7826	0.8143	0.8501	0.6742	0.7200	0.7992	0.0549
DF1+DF2+CF+WF1+WF2+WF3	0.8494	0.7854	0.8160	0.8517	0.6749	0.7531	0.8012	0.0569
DF1+DF2+CF+WF1+WF2+WF3+HF	0.8509	0.7796	0.8137	0.8695	0.6893	0.7690	0.8031	0.0588

*Inc is increment compared with baseline.

**Table 10:** Performance with training datasets of various sizes using automatically predicted medical concepts.

	Explicit recognizer			Implicit recognizer			Micro	INC
Data Size	P	R	F	P	R	F	F	
50	0.8314	0.7308	0.7779	0.8403	0.5817	0.6875	0.7568	/
100	0.8456	0.7527	0.7965	0.8515	0.6401	0.7308	0.7810	0.0242
150	0.8473	0.7628	0.8028	0.8619	0.6531	0.7431	0.7888	0.0320
200	0.8501	0.7736	0.8100	0.8643	0.6764	0.7589	0.7981	0.0413
250	0.8518	0.7774	0.8129	0.8678	0.6852	0.7658	0.8019	0.0451
300	0.8509	0.7796	0.8137	0.8695	0.6893	0.7690	0.8031	0.0463

*Inc is increment compared with baseline.

Note that in Table 6 and 9, the increments achieved by each feature combinations are compared with baseline, rather than with the previous combination.

## Results

**Table 5** shows that different features had varying effects on the two recognizers. As we expected, the ontological feature based on the anatomy dictionary (DF1) contributed greatly to the performance of the explicit anatomical recognizer, while its effect on implicit entities was negligible. The world knowledge features (WF1, WF2 and WF3) brought improvement to both the implicit and explicit anatomical recognizers. In particular, their use resulted in a significant improvement in the recognition of implicit anatomical entities. Though less significant, WF2 and WF3 unexpectedly improved the performance of the explicit anatomical entity recognizer. The improvement in precision was much greater than recall for explicit entities, while the opposite is the case for implicit entities. This shows that the WF2 and WF3 features are treated as negative evidence for explicit entities and positive evidence for implicit entities.

As shown in **Table 6**, the combination of different features contributed to improving the overall performance of the whole recognizer. The best performance of F1 0.8978 was achieved when all features were combined.

From the results shown in **Table 7**, the recall of the system significantly increased when a lager dataset was used. Precision also increases with the size of the dataset, but to a lesser extent than recall.

In order to exclude the positive influence of the gold standard of I2B2 annotation, we used the results produced by the multi-class recognizer which we developed for I2B2 challenges. **Tables 8**, **9**, **and** **10**, show the results, which illustrate the same trends that were observed in the previous tables. The performance achieved demonstrates that our method can be applied within a real-world setting, while achieving acceptable levels of performance.

## Discussion

The proposed framework, which uses three entity recognizers (i.e. multi-class recognizer, explicit anatomical entity recognizer and implicit anatomical entity recognizer) in a hierarchical fashion, shows satisfactory performance for anatomical entity recognition. While the features based on the dictionary of anatomical entity expressions greatly improved the performance on explicit anatomical entities, they do not enhance the performance on implicit anatomical entities. Our framework uses two external knowledge resources, i.e., Wikipedia and WordNet, to aid in the recognition of both explicit and implicit anatomical entities. The addition of features based on these external resources results in increments between 2.14% and 2.67% over the baseline features, in terms of overall performances.

Dictionary-matching method can efficiently recognize common explicit anatomical entities in medical texts (e.g. lung, heart), as the increment of DF shows in the tables. Although simply dictionary-matching can hardly match the implicit anatomical named entities, this method still is an effective way to recognize common anatomical entities in World Knowledge features.

The fact that the features derived from these external knowledge resources significantly enhances the performance of the system demonstrates that inferences based on medical domain knowledge play an important role in entity recognition. Our hierarchical framework is one of possible solution for exploiting domain knowledge. A further solution would be to construct more structured knowledge bases, similar to Freebase [40] or Yago [41], but for the medical domains, instead of on-the-fly recognition of candidates of implicit anatomical entities. However, construction of such structured knowledge bases would be costly and would involve manual intervention. Considering the ever-expanding domain knowledge, our approach of on-the-fly recognition of association between implicit entities and anatomical entities has its own advantages.

The improvement obtained by combining Wikipedia and WordNet features (i.e. WF2 and WF3) is not great, compared with the improvement obtained by the individual use of these features. This may imply that the two resources have significant overlaps.

Overall performance improvement obtained by all features combined was 5.29% (when the I2B2 gold standard annotations were used) and 5.08% (when the results of the multi-class recognizers were used).

Our framework considers only one step inferences, but cannot currently handle deeper inferences. For example, “dm” (diabetes mellitus) is annotated in the gold standard text as an implicit anatomical entity. This is because “dm” is caused by shortness of insulin, which in turn is caused by abnormities in the pancreas. Such two-step inferences cannot be handled correctly by our framework. This is because the definition of “dm” in Wikipedia refers to “insulin”, but does not contain any mentions of pancreas in the first three sentences.

We also encounter other problems that are commonly faced when processing medical records. One such problem is that, since the language used in medical records is less formal than language in newspapers or scientific publications, non-standard abbreviations frequently appear. For example, “cabgx4” and “R-PDA”, which stand for “coronary artery bypass graft by 4 times” and “right posterior descending artery” respectively, were annotated as explicit anatomical entities. The dictionary matching feature cannot be derived from such abbreviations, unless a system is developed which deals with such abbreviations adequately. Abbreviations or non-standard shortened forms in medical records cause difficulties not only in dictionary matching but also in accessing to their corresponding entries in the knowledge sources. For example, the word “extremity” has several abbreviations like “extrem” ”, “ext”, “ue” (upper extremity), and “le” (lower extremity), and not all abbreviations of this word are registered in the external resources.

The problem of ambiguity, which is a challenge faced by all named entity recognition tasks, also occurred in our task. For example, a significant number of errors was caused by the word “back”, since it occurred in the corpus both as an explicit anatomical entity and as an adverbial.

## Conclusions

This paper described a system based on a CRF model baseline, augmented with features based on external resources and a hierarchical framework to accomplish the objective of automatic anatomical NER. Whilst NER that includes recognition of explicit anatomical entities has been widely studied and can achieve high quality results, the task of implicit anatomical entity extraction, which is more complex and demanding, differentiates our work from others. A hierarchical framework was proposed especially for the task of implicit anatomical entity recognition and the best result achieved, i.e. an F1 score of 0.8537, demonstrates the effectiveness of our approach. A key element of our approach is the use of external resources, i.e., Wikipedia and WordNet, as a means to identify implicit anatomical entities, using anatomical information as cues. The result of experiments that employ world knowledge from these resources as demonstrates their helpfulness.

Based on the encouraging results achieved by our method in the recognition of anatomical entity from discharge summaries, our future work will involve extending the application areas as well as improving the results achieved. As the first step to extracting anatomical information from narrative medical records, this task achieves the goal of extracting anatomical entities, both explicit and implicit. To further structure and utilize anatomical information, we will carry out normalization of anatomical entities to map them to corresponding body components, thus enabling wider application of the extracted information.

Our results clearly demonstrate the crucial nature of external resources in improving anatomical entity recognition. Since further web-based sources contain a wealth of potentially useful information, we aim to introduce a greater number of such resources into our framework to improve the performance of the recognizers.

Since we mainly focus on anatomical entities in this work, we directly used gold standard coreference data. In the subsequent work, we will enhance our framework by adding the systems we already developed to automatically generate coreference information, which will be incorporated into the framework as additional features.

## References

[1] Spyns P (1996) Natural Language Processing in Medicine: An Overview. Methods Inf Med 35: 285–301. 9019092

[2] Friedman C, Shagina L, Lussier Y, Hripcsak G (2004) Automated Encoding of Clinical Documents Based in Natural Language Processing. J Am Med Inform Assoc 11: 392–402. 15187068 10.1197/jamia.M1552 PMC516246

[3] Meystre SM, Haug PJ (2005) Comparing Natural Language Processing Tools to Extract Medical Problems from Narrative Text. AMIA Annu Symp Proc 525–9. 16779095 PMC1560561

[4] Meystre SM, Haug PJ (2006) Natural Language Processing to Extract Medical Problems from Electronic Clinical Documents: Performance Evaluation. J Biomed Inform 39 6: 589–99. 16359928 10.1016/j.jbi.2005.11.004

[5] Sager N, Lyman M, Bucknall C, Nhan N, Tick LJ (1994) Natural Language Processing and the Representation of Clinical data. J Am Med Inform Assoc 2: 142–60. 10.1136/jamia.1994.95236145 PMC116193 7719796

[6] Pyysalo S, Ananiadou S (2014) Anatomical Entity Mention Recognition at Literature Scale. Bioinformatics 30 6: 868–875. 24162468 10.1093/bioinformatics/btt580 PMC3957068

[7] Uzuner O, South BR, Shen S, DuVall SL (2011) 2010 i2b2/VA Challenge on Concepts, Assertions, and Relations in Clinical Text. J Am Med Inform Assoc 18: 552–556. 21685143 10.1136/amiajnl-2011-000203 PMC3168320

[8] CRF + +. Available: http://crfpp.googlecode.com/svn/trunk/doc/index.html. Accessed 2014 Sep 3.

[9] John L, Andrew M, Fernando P (2001) Conditional Random Fields: Probabilistic Models for Segmenting and Labeling Sequence Data. Proceedings of the 18th international Conference on Machine Learning (ICML 2001). Williamstown, MA, USA).

[10] Li X, Wang YY, Acero A (2009) Extracting Structured Information from User Queries with Semi-supervised Conditional Random Fields. Proceedings of the 32nd ACM Special Interest Group on Information Retrieval (SIGIR 2009). Boston, MA, USA.

[11] UMLS Knowledge Base. Available: http://www.nlm.nih.gov/research/umls. Accessed 2014 Sep 3.

[12] Mesh Knowledge Base. Available: http://www.ncbi.nlm.nih.gov/mesh. Accessed 2014 Sep 3.

[13] RadLex Knowledge Base. Available: http://www.radlex.org/. Accessed 2014 Sep 3.

[14] BodyParts3D Knowledge Base. Available: http://lifesciencedb.jp/bp3d/?lng=en. Accessed 2014 Sep 3.

[15] Schwartz AS, Hearst MA (2003) A Simple Algorithm for Identifying Abbreviation Definitions in Biomedical Text. Pacific Symposium on Biocomputing 8: 451–462. 12603049

[16] Hripcsak G, Friedman C, Alderson PO, DuMouchel W, Johnson SB, et al (1995) Unlocking Clinical Data from Narrative Reports: A Study of Natural Language Processing. Ann Intern Med 122: 681–8. 7702231 10.7326/0003-4819-122-9-199505010-00007

[17] Friedman C, Alderson PO, Austin JH, Cimino JF, Johnson SB (1994) A General Natural–language Text Processor for Clinical Radiology. J Am Med Inform Assoc 1: 161–74. 7719797 10.1136/jamia.1994.95236146 PMC116194

[18] Friedman C, Shagina L, Lussier Y, Hripcsak G (2004) Automated Encoding of Clinical Documents Based on Natural Language Processing. J Am Med Inform Assoc 11: 392–402. 15187068 10.1197/jamia.M1552 PMC516246

[19] Hripcsak G, Austin JH, Alderson PO, Friedman C (2002) Use of Natural Language Processing to Translate Clinical Information from a Database of 889,921 Chest Radiographic Reports. Radiology 224: 157–63. 12091676 10.1148/radiol.2241011118

[20] The Third i2b2 challenge. Available: https://www.i2b2.org/NLP/Medication/. Accessed 2014 Sep 3.

[21] The Fourth i2b2/VA challenge. Available: https://www.i2b2.org/NLP/Relations/. Accessed 2014 Sep 3.

[22] Ananiadou S, Thompson P, Nawaz R, McNaught J, Kell DB (2014) Event Based Text Mining for Biology and Functional Genomics. Briefings in Functional Genomics In press. 10.1093/bfgp/elu015 PMC4499874 24907365

[23] Rosse C, Mejino JL (2003) A Reference Ontology for Biomedical Informatics: The Foundational Model of Anatomy. Journal of Biomedical Informatics 36: 478–500. 14759820 10.1016/j.jbi.2003.11.007

[24] Rosse C, Mejino JL (2008) The Foundational Model of Anatomy Ontology. Anatomy Ontologies for Bioinformatics 59–117.

[25] Jin-Dong K, Tomoko O, Yoshimasa T, Yuka T, Nigel C (2004) Introduction of the Bio-entity Recognition Task at JNLPBA. Proceeding of JNLPABA 2004.

[26] Martin G, Goran N, Casey MB (2010) An Exploration of Mining Gene Expression Mentions and Their Anatomical Locations from Medical Text. BioNLP 72–80.

[27] Naderi N, Kappler T, Baker CJ, Witte R (2011) OrganismTagger: Detection, Normalization and Grounding of Organism Entities in Biomedical Documents. Bioinformatics 27 19: 2721–9. 21828087 10.1093/bioinformatics/btr452

[28] Junichi K, Kentaro T (2007) Exploiting Wikipedia as External Knowledge for Named Entity Recognition. Proceeding of the 2007 Joint Conference on Empirical Methods in Natural Language Processing and Computational Natural Language Learning (EMNLP-CoNLL 2007). Prague, Czech Republic. pp. 698–707.

[29] Cucerzan S (2007) Large-scale Named Entity Disambiguation Based on Wikipedia Data. Proceeding of the 2007 Joint Conference on Empirical Methods in Natural Language Processing and Computational Natural Language Learning (EMNLP-CoNLL 2007). Prague, Czech Republic. pp. 708–716.

[30] Rink B, Harabagie S, Roberts K (2011) Automatic Extraction of Relations between Medical Concepts in Clinical Texts. J Am Med Inform Assoc 18: 594–600. 21846787 10.1136/amiajnl-2011-000153 PMC3168312

[31] Yan X, Jiahua L, Jiajun W, Yue W, Zhuowen T, et al (2012) A Classification Approach to Coreference in Discharge Summaries: 2011 i2b2 challenge. J Am Med Inform Assoc 19: 897–905. 22505762 10.1136/amiajnl-2011-000734 PMC3422828

[32] Yan X, Yue W, Jiahua L, Zhuowen T, Jian-Tao S, et al (2012) Suicide Note Sentiment Classification: A Supervised Approach Augmented by Web Data. Biomed Inform Insights 5: 31–41. 22879758 10.4137/BII.S8956 PMC3409493

[33] Wikipedia. Available: http://www.wikipedia.org/. Accessed 2014 Sep 3.

[34] WordNet. Available: http://wordnet.princeton.edu/. Accessed 2014 Sep 3.

[35] Zuo MX (2009) Human Anatomy and Physiology. Beijing, China: Higher Education Press. 11 p.

[36] Carletta J (1996) Agreement on Classification Tasks: The Kappa Statistics. Computational Linguistics 22: 249–254.

[37] Enju Paser. Available: http://www.nactem.ac.uk/tsujii/enju/. Accessed 2014 Sep 3.

[38] Stanford Parser. Available: http://www-nlp.stanford.edu/software/lex-parser.shtml. Accessed 2014 Sep 3.

[39] Powers D (2007) Evaluation: From Precision, Recall and F-Factor to ROC, Informedness, Markedness & Correlation. Technical Report 7:001.

[40] Freebase. Available: http://www.freebase.com/. Accessed 2014 Sep 3.

[41] Yago. Available: http://www.mpi-inf.mpg.de/departments/databases-and-information-systems/research/yago-naga/yago/. Accessed 2014 Sep 3.

